# The association of ideal cardiovascular health and its change with subclinical atherosclerosis according to glucose status: A prospective cohort study

**DOI:** 10.1111/1753-0407.70007

**Published:** 2024-10-10

**Authors:** Xiaojing Jia, Yilan Ding, Chunyan Hu, Hong Lin, Lin Lin, Xueyan Wu, Hongyan Qi, Shuangyuan Wang, Ruizhi Zheng, Jie Zheng, Min Xu, Yu Xu, Tiange Wang, Zhiyun Zhao, Yuhong Chen, Mian Li, Guang Ning, Weiqing Wang, Weiguo Hu, Yufang Bi, Jieli Lu

**Affiliations:** ^1^ Department of Endocrine and Metabolic Diseases, Shanghai Institute of Endocrine and Metabolic Diseases, Ruijin Hospital Shanghai Jiao Tong University School of Medicine Shanghai China; ^2^ Shanghai National Clinical Research Center for Metabolic Diseases, Key Laboratory for Endocrine and Metabolic Diseases of the National Health Commission of the PR China, Shanghai Key Laboratory for Endocrine Tumor, State Key Laboratory of Medical Genomics, Ruijin Hospital Shanghai Jiao Tong University School of Medicine Shanghai China; ^3^ Department of Geriatrics, Medical Center on Aging, Ruijin hospital Shanghai Jiao Tong University School of Medicine Shanghai China

**Keywords:** cardiovascular health, hyperglycemia, Life's Essential 8, subclinical atherosclerosis

## Abstract

**Background:**

An updated definition was developed to better evaluate cardiovascular health (CVH). We aimed to investigate whether optimal or improvement of six CVH metrics defined by new Life's Essential 8 (LE8) may counteract the risk of subclinical atherosclerosis among patients with hyperglycemia.

**Methods:**

We conducted a prospective analysis of 5225 participants without prior cardiovascular diseases, of whom 4768 had complete data on CVH change. Subjects with CVH scores of 0–49, 50–79, and 80–100 points were categorized as having low, moderate, or high CVH, respectively. Subclinical atherosclerosis was evaluated by brachial‐ankle pulse wave velocity, pulse pressure and albuminuria, both separately and in combination.

**Results:**

Of the 5225 participants, 1937 (37.1%) had normal glucose regulation, while 3288 (62.9%) had hyperglycemia. The multivariable‐adjusted odds ratio (OR) for composite subclinical atherosclerosis was 2.34 (95% confidence interval [CI], 1.88–2.91), 1.43 (95% CI, 1.21–1.70), and 0.74 (95% CI, 0.46–1.18), for participants with hyperglycemia who had low, moderate, or high overall CVH scores, respectively, compared with participants with normal glucose regulation. In addition, compared with those with stable CVH and normal glucose regulation, participants who exhibited greater improvements in overall CVH from 2010 to 2014 had a reduced risk of composite subclinical atherosclerosis with an OR of 0.72 (95% CI, 0.53–0.98) for those with normal glucose regulation, and 1.13 (95% CI, 0.87–1.48) for those with hyperglycemia.

**Conclusions:**

The novel defined CVH using six metrics was inversely associated with subsequent risk of subclinical atherosclerosis. Both the status of CVH and its changes modified the relationship between hyperglycemia and subclinical atherosclerosis.

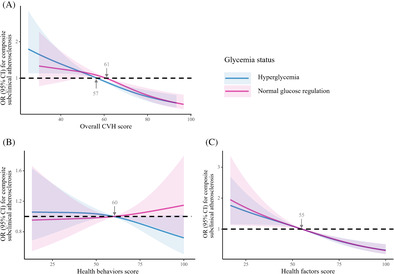

## Graphical abstract

Highlights

- Participants with hyperglycemia who had high score of CVH exhibited no significant excess risk of subclinical atherosclerosis.
- Improvement in CVH during a median follow‐up of 4.3 years showed a beneficial effect on reduced risk of subclinical atherosclerosis in individuals with hyperglycemia, compared with those who had normal glucose regulation and stable CVH.
- Promoting the adherence to, and the improvement of, both healthy behaviors and factors was a key strategy to tackle the onset of atherosclerosis.

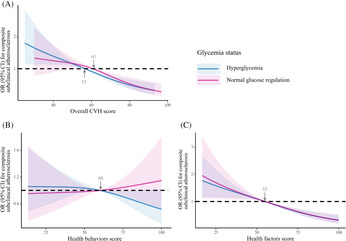

## 1 INTRODUCTION

Cardiovascular health (CVH) was initially proposed by American Heart Association (AHA) in 2010 based on seven healthy lifestyle behaviors and ideal metabolic measures, and potential evidence has substantiated its correlation with a broad spectrum of health outcomes.^1^, ^2^ In June 2022, the AHA updated the definitions and scoring of the original “Life's Simple 7” (LS7) CVH metrics and introduced a new sleep metric, employing on a more continuous scale to increase the sensitivity to inter‐individual differences and changes over time.^3^ This revised framework, now termed “Life's Essential 8” (LE8) CVH encompasses four behavioral metrics (diet, physical activity, nicotine exposure, and sleep health) and four metabolic factors metrics (body mass index [BMI], non‐high‐density lipoprotein cholesterol [non‐HDL‐C] level, blood glucose, and blood pressure [BP]). The new LE8 was highly correlated with the LS7 score and has been employed in a recent study to quantify the CVH levels in the US population.^4^ Epidemiological evidence on the relationship between CVH using the new LE8 metrics and cardiovascular events was limited.

The epidemic proportion of hyperglycemia worldwide and especially in China was high, imposing a major threat to public health. According to estimates from the International Diabetes Federation, approximately 1 in 10 adults were affected by diabetes (536.6 million), with an additional 10.6% of adults worldwide (541 million) experiencing impaired glucose tolerance.^5^ This escalating issue is further exacerbated by the well‐established link between hyperglycemia and an elevated risk of cardiovascular morbidity and mortality,^6^, ^7^ and extensive evidence indicates that both diabetes and prediabetes confer a significant excess risk for cardiovascular disease (CVD),^8^, ^9^, ^10^ as well as subclinical atherosclerosis.^11^, ^12^ Given the great prevalence of these high‐risk individuals, it is imperative to recognize that a considerable burden of related CVD events is imminent. To effectively address this challenge, comprehensive recommendations and multifactorial interventions are needed.

Meanwhile, subclinical atherosclerosis, manifested by elevated brachial‐ankle pulse wave velocity (baPWV), pulse pressure (PP) or albuminuria, is the early stage of CVD and a quite important turning point,^13^, ^14^, ^15^ as the intervening measures can be effectively implemented at this stage to prevent the onset of clinical events. Previous population‐based studies indicated that subjects with higher numbers of ideal LS7 CVH metrics were less likely to have atherosclerosis in both western and Chinese populations.^16^, ^17^ Besides, a previous study has shown that participants with prediabetes or diabetes who had 5 or more ideal LS7 CVH metrics appeared to have a lower or no increased risk of CVD compared with normal glucose regulation.^18^ These findings emphasized the importance of promoting CVH in the prevention of CVD among patients with diabetes or prediabetes. The association of hyperglycemia and risk of early stage of CVD as measured by subclinical atherosclerosis has not been investigated by using the new LE8 score. The question remains unanswered is whether improvements in LE8 CVH score may yield cardiovascular benefits among patients with hyperglycemia.

To fill these critical knowledge gaps, in a community‐based cohort of middle‐aged and elderly adults, we conducted a prospective analysis to investigate the associations of the new CVH status and its improvement over time with the risk of subclinical atherosclerosis among individuals with hyperglycemia compared with individuals with normal glucose regulation.

## 2 METHODS

### 2.1 Study design and populations

We conducted this prospective study in a community‐based population in Jiading District, Shanghai, China, which has been described in detail elsewhere.^19^ Briefly, a total of 10 375 participants (aged 40 years or above) were recruited between March and August 2010, during which time they provided responses on a standard questionnaire through face‐to‐face interviews and underwent relevant clinical examinations. Follow‐up visits were carried out between August 2014 through May 2015. Measurements of subclinical atherosclerosis or other clinical metrics were performed using the same rigid protocols as those at baseline.

**FIGURE 1:**
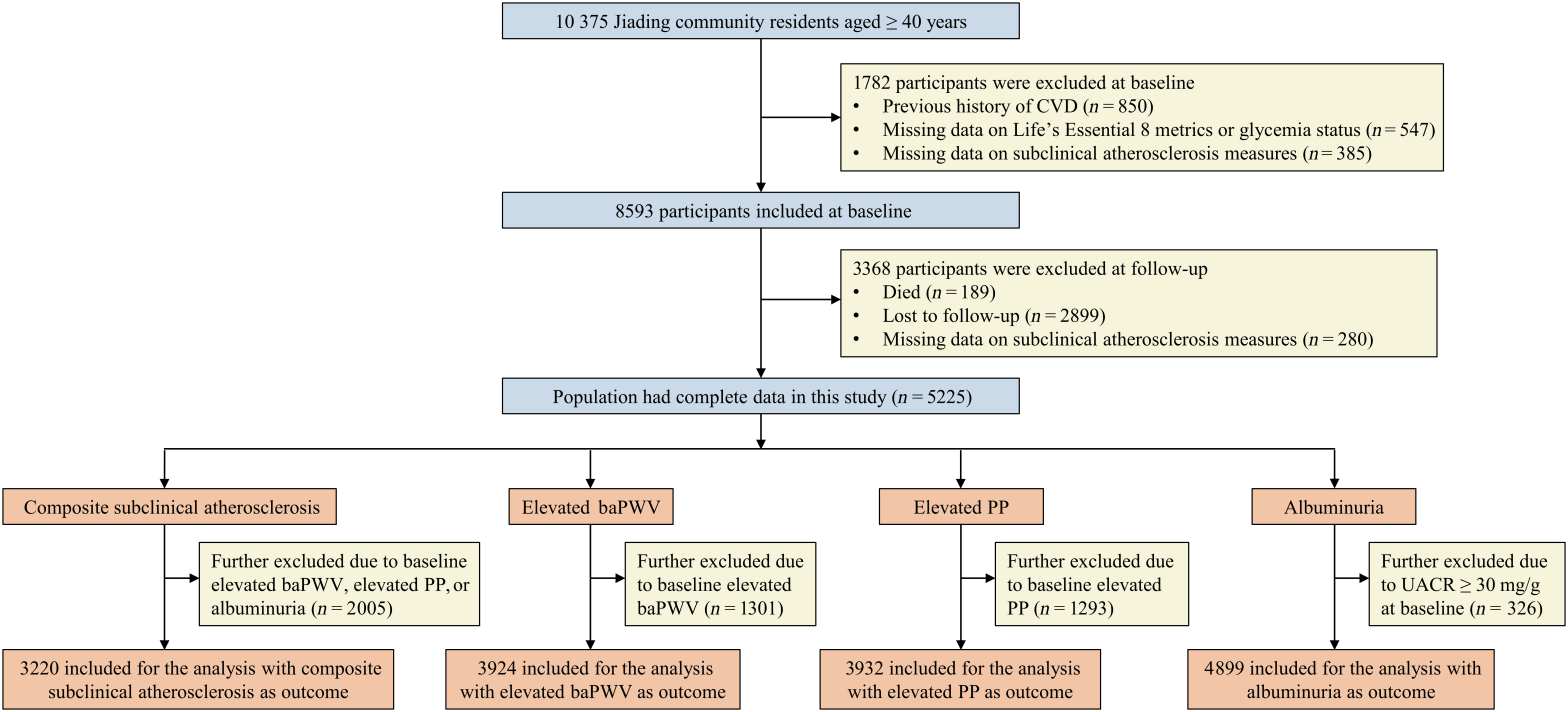
Flowchart of study population. baPWV, brachial‐ankle pulse wave velocity; CVD, cardiovascular disease; PP, pulse pressure; UACR, urinary albumin/creatinine ratio.

For current analysis, participants who had complete information on CVH metrics, glycemia status, and subclinical atherosclerosis measures and were free from CVD at baseline were included (*n* = 8593). We further excluded those who died (*n* = 189), were not invited to attend follow‐up (*n* = 2899) or had missing data on subclinical atherosclerosis measures at follow‐up (*n* = 280). Finally, there were 5225 participants included in the prospective analysis. The flowchart of the participant selection process is shown in Figure 1. For measuring and monitoring the change in CVH between 2010 and 2014, participants with incident CVD (*n* = 138) or missing data on CVH metrics at follow‐up visit (*n* = 319) were further excluded, leaving 4768 individuals.

The study protocol was approved by the Ethics Committee of Ruijin Hospital, Shanghai Jiao Tong University School of Medicine. All participates signed written informed consent and took a comprehensive physical examination and interview.

### 2.2 Data collection

Information on demographic characteristics, lifestyle factors (physical activity, cigarette smoking, sleep habit, and alcohol consumption), and current use of medications were collected by trained interviewers using a standard detailed questionnaire at baseline and follow‐up visits. Current alcohol drinkers were defined as those who consumed alcohol regularly in the past 6 months. Education attainment was categorized as less than high school and high school or more. The International Physical Activity Questionnaire (IPAQ) was used to collect information on intensity, duration and frequency of physical activity.^20^

Body weight and height were measured according to a standard protocol, and BMI was calculated as weight in kilograms divided by height in meters squared. Three BP measurements were consecutively collected on the non‐dominant arm of each seated participant after a ≥5‐min rest using an automated electronic device (OMRON Model HEM‐752 FUZZY, Omron Company, Dalian, China). The readings were averaged for analysis. PP was defined as systolic BP (SBP) minus diastolic BP (DBP).

BaPWV was measured by trained physicians using Colin VP‐1000 (model BP203RPEII, form PWV/ABI (ankle‐brachial index); Omron Colin Medical Instruments, Tokyo, Japan) in two visits. Pulse waves were obtained with cuffs placed on both the right and left upper arms and ankles after participants had rested for 10 to 15 min. Transit time, the time interval between the ipsilateral initial increase in brachial and tibial waveforms, and transit distance between the ipsilateral arm and ankle were measured. The values of right and left baPWV were calculated as the transit distance divided by the transit time and the larger one was used for analysis.

All participants underwent a 75‐g oral glucose tolerance test (OGTT) after an overnight fast of at least 10 h, and blood samples were collected at 0 and 2 h during the test. Fasting and OGTT 2‐h plasma glucose concentrations were evaluated using the glucose oxidase method. Glycated hemoglobin A1c (HbA_1c_) was determined within 4 weeks after collection by using the method of high‐performance liquid chromatography (VARIANT™ II and D‐10™ Systems, BIO‐RAD, Hercules, CA, USA). Levels of total cholesterol (TC) and HDL‐C were measured with an autoanalyzer (Modular E170; Roche, Basel, Switzerland) in Shanghai Institute of Endocrine and Metabolic Diseases, which is certified by the College of American Pathologists (CAP). Non‐HDL‐C was calculated by subtracting HDL‐C from TC.

A first‐voided, early‐morning spot urine sample was collected from each participant to measure urinary albumin (g/L) and creatinine (mmol/L) using the immunoturbidimetric method (Beijing Atom High‐Tech, Beijing, China) and the Jaffe's kinetic method on an automatic analyzer (Hitachi 7600–020, Tokyo, Japan), respectively. Urinary albumin/creatinine ratio (UACR) was calculated as the urinary albumin concentration divided by the urinary creatinine concentration and is expressed in milligrams per gram.

### 2.3 Assessment of baseline glucose tolerance status

Baseline glucose tolerance status were defined according to the American Diabetes Association 2010 criteria.^21^ Diabetes was defined as fasting plasma glucose level of at least 126 mg/dL (7.0 mmol/L), OGTT 2‐h plasma glucose level of at least 200 mg/dL (11.1 mmol/L), HbA_1c_ level of at least 6.5%, or by a self‐reported previous diagnosis by health care professionals. In participants without diabetes, normal glucose tolerance was defined as fasting plasma glucose concentration of less than 100 mg/dL (5.6 mmol/L), OGTT 2‐h plasma glucose concentration of less than 140 mg/dL (7.8 mmol/L), and HbA_1c_ of less than 5.7%; prediabetes was defined as fasting plasma glucose between 100 mg/dL (5.6 mmol/L) and less than 126 mg/dL (7.0 mmol/L), or OGTT 2‐h plasma glucose between 140 mg/dL (7.8 mmol/L) and less than 200 mg/dL (11.1 mmol/L), or HbA_1c_ between 5.7% and less than 6.5%. Concisely, participants with diabetes or prediabetes were considered in hyperglycemia status.

### 2.4 Quantification of CVH

Definitions and scoring for the component metrics of CVH, including three health behaviors (physical activity, nicotine exposure, and sleep health) and three health factors (BMI, non‐HDL‐C, and BP) (Table S1), are following the new AHA algorithm.^3^ Blood glucose metric was not included in the calculation of LE8 CVH score, as this study was designed to assess the associations of ideal CVH with subsequent development of subclinical atherosclerosis among participants with hyperglycemia as compared with participants with normal glucose regulation. Besides, dietary metric was not included due to the not available data. CVH behavior (or health factor) was calculated by summing the scores for behaviors (or factors) metrics together and dividing the total by 3. Overall, CVH was calculated as the unweighted average of all six component metric scores. Individual or combined CVH score across a range of 0 to 100 was classified as low (0 to 49 points), moderate (50 to 79 points), and high (80 to 100 points).

Change in CVH between 2010 and 2014 was examined in participants having six metrics at both time points. We also calculated a score representing change in overall CVH from 2010 to 2014. The score was further categorized as gained in those with more than 5 points, stable in those with −5 to 5 points, and lost in those with less than −5 points.

### 2.5 Outcome assessment

The investigated outcomes were elevated baPWV, elevated PP, albuminuria, and their combination as the composite subclinical atherosclerosis. Elevated baPWV and elevated PP were defined as the upper quartile of corresponding values at baseline or follow‐up. Incident albuminuria was defined as UACR ≥30 mg/g (3.4 mg/mmol). Incident CVD was defined as the first instance of myocardial infarction, stroke, or heart failure.

### 2.6 Statistical analysis

Baseline characteristics of participants with normal glucose regulation or hyperglycemia were summarized as means with standard deviation (SD) or median (interquartile range) for continuous variables and percentages for categorical variables.

We compared the incident risk of subclinical atherosclerosis between hyperglycemia with different CVH statuses and the normal glucose regulation (reference group) to observe whether adherence to healthy CVH could offset the risk of subclinical atherosclerosis. Logistic regression models were used to calculate odds ratios (ORs) and 95% confidence intervals (CIs) for incident subclinical atherosclerosis (separate/composite), with multivariable adjustment for age, sex, education attainment, and current drinking status. In the analysis of each component of the CVH, we split our cohort into three groups: hyperglycemia with high CVH, hyperglycemia with low‐to‐moderate CVH, and normal glucose regulation (reference group). And we further adjusted for all other five CVH metrics. In the analysis of combined CVH (health behaviors, health factors, and overall CVH), we separated those with a high, moderate, or low CVH among the participants with hyperglycemia and compared with normal glucose regulation (reference group). Health behaviors metrics and health factors metrics were further mutually adjusted.

As a next analysis, we stratified participants into subgroups according to baseline glycemia status. Among participants with normal glucose regulation or hyperglycemia, we analyzed the association between subclinical atherosclerosis risk and categorical (low, moderate, high) or continuous (per 10‐point increment) score of combined CVH. Restricted cubic splines with three knots at the 5th, 50th, and 95th percentiles were further used to fit and model such relationship.

Moreover, to explore whether the impact of change in CVH on subclinical atherosclerosis varied by baseline glycemia status, the ORs of subclinical atherosclerosis per 10‐point increment in the changes in overall CVH were estimated. Besides, we created a variable with six categories, which combines baseline glycemia status (normal glucose regulation, hyperglycemia) with CVH change (lost, stable, and gained) to investigate their joint effect on risk of subclinical atherosclerosis, by using the group with stable CVH and normal glucose regulation as the reference category.

A 2‐sided *p* value of less than 0.05 was considered statistically significant. We used SAS version 9.4 (SAS Institute, Cary, NC) to conduct statistical analyses and R version 4.0.5 (http://www.r-project.org) to plot restricted cubic splines and forest maps.

## 3 RESULTS

Of the 5225 participants, 1937 (37.1%) participants had normal glucose regulation and 3288 (62.9%) had hyperglycemia at baseline. Mean (SD) age ranged from 55.2 (8.4) years to 58.7 (8.3) years. Baseline characteristics of the study participants according to baseline glucose tolerance status are shown in Table 1. Compared with participants with normal glucose regulation, participants with hyperglycemia were older, had higher proportions of men, had lower level of education attainment, were more likely to have higher values of subclinical atherosclerosis measures, and had lower scores of health factors or overall CVH.

**TABLE 1:** Baseline characteristics of participants with normal glucose regulation and hyperglycemia.

Baseline characteristic	Normal glucose regulation	Hyperglycemia
	(*N* = 1937)	(*N* = 3288)
Age, years	55.2 ± 8.4	58.7 ± 8.3
Male sex, *n* (%)	706 (36.5)	1216 (37.0)
High school or above education, *n* (%)	460 (23.8)	603 (18.4)
Current alcohol drinker, *n* (%)	401 (20.7)	650 (19.8)
baPWV, cm/s	1495.7 ± 311.5	1643.2 ± 351.4
PP, mmHg	53.9 ± 14.3	60.1 ± 15.7
UACR, mg/g	4.3 (2.5–7.3)	5.2 (3.0–9.9)
Overall CVH score	59.4 ± 14.0	54.7 ± 13.5
Health behaviors score	58.8 ± 19.7	59.6 ± 20.6
Physical activity score	21.3 ± 40.6	21.6 ± 40.9
Nicotine exposure score	67.2 ± 40.1	69.2 ± 39.6
Sleep health score	87.9 ± 21.3	88.0 ± 20.8
Health factors score	60.1 ± 19.8	49.8 ± 18.4
Body mass index score	71.5 ± 22.9	63.7 ± 23.1
Blood lipids (non‐HDL‐C) score	62.3 ± 28.3	53.5 ± 28.3
Blood pressure score	46.5 ± 34.5	32.2 ± 30.6

*Note*: Data were presented as means (standard deviations) or medians (interquartile ranges) for continuous variables or numbers (percentages) for categorical variables.

Abbreviations: BaPWV, brachial‐ankle pulse wave velocity; CVH, cardiovascular health; non‐HDL‐C, non‐high‐density lipoprotein cholesterol; PP, pulse pressure; UACR, urinary albumin/creatinine ratio.

During a median follow‐up period of 4.3 years, we documented 1291 (40.1%) cases of composite subclinical atherosclerosis, 389 (7.9%) cases of albuminuria, 980 (25.0%) cases of subclinical atherosclerosis reflected by elevated baPWV and 975 (24.8%) cases of subclinical atherosclerosis reflected by elevated PP. As shown in Table 2, compared with reference group (participants with normal glucose regulation), participants with hyperglycemia who had 1 of these CVH metrics exhibited nonsignificant higher risk for composite subclinical atherosclerosis: high physical activity score (OR, 1.26; 95% CI, 0.98–1.61), high BMI score (OR, 1.20; 95% CI, 0.94–1.54), and high blood lipids score (OR, 1.27; 95% CI, 0.99–1.62). Besides, there was a significantly decreased risk in participants with hyperglycemia who had high BP score (OR, 0.65; 95% CI, 0.48–0.90). Compared with participants with normal glucose regulation, among participants with hyperglycemia, the OR for composite subclinical atherosclerosis gradually decreased from 2.34 (95% CI, 1.88–2.91) to 1.43 (95% CI, 1.21–1.70) and 0.74 (95% CI, 0.46–1.18), respectively, in those with low, moderate and high overall CVH scores. A similar pattern was observed in the associations of health behaviors and health factors with composite subclinical atherosclerosis. Compared with participants with normal glucose regulation, the OR (95% CI) was 1.65 (1.31–2.08), 1.39 (1.16–1.66) and 1.19 (0.89–1.59) in participants with hyperglycemia with low, moderate, and high health behaviors scores, and 2.26 (1.86–2.75), 1.42 (1.18–1.70), and 0.77 (0.55–1.07) in participants with hyperglycemia with low, moderate, and high health factors scores, respectively. The data for incident subclinical atherosclerosis reflected by elevated baPWV, elevated PP, or albuminuria showed a similar trend as those for composite subclinical atherosclerosis (Table 2).

**TABLE 2:** Odds ratio (95% CI) of subclinical atherosclerosis according to individual and overall cardiovascular health metric among participants with hyperglycemia, as compared with participants with normal glucose regulation.^a^

Category	Composite subclinical atherosclerosis		Elevated baPWV		Elevated PP		Albuminuria	
	Case/*N*	OR (95% CI)^b^	Case/*N*	OR (95% CI)^b^	Case/*N*	OR (95% CI)^b^	Case/*N*	OR (95% CI)^b^
Normal glucose regulation	444/1412	1.00 (ref)	288/1628	1.00 (ref)	305/1616	1.00 (ref)	100/1852	1.00 (ref)
Hyperglycemia								
High physical activity score	168/383	1.26 [0.98–1.61]	151/488	1.53 [1.20–1.97]	138/489	1.27 [0.99–1.62]	72/645	1.77 [1.28–2.45]
High nicotine exposure score	617/1262	1.41 [1.18–1.69]	496/1650	1.42 [1.17–1.71]	511/1632	1.26 [1.05–1.51]	228/2225	1.44 [1.11–1.86]
High sleep health score	655/1423	1.39 [1.18–1.64]	537/1829	1.42 [1.19–1.69]	534/1829	1.31 [1.10–1.56]	225/2403	1.45 [1.13–1.86]
High BMI score	164/403	1.20 [0.94–1.54]	135/495	1.30 [1.01–1.69]	127/512	1.10 [0.86–1.42]	60/641	1.46 [1.04–2.06]
High blood lipids score	169/406	1.27 [0.99–1.62]	139/492	1.42 [1.10–1.82]	126/494	1.21 [0.94–1.56]	49/612	1.32 [0.92–1.90]
High blood pressure score	67/257	0.65 [0.48–0.90]	37/267	0.68 [0.46–1.01]	21/277	0.30 [0.19–0.48]	13/266	0.92 [0.51–1.68]
Health behaviors score								
Low (0–49)	241/528	1.65 [1.31–2.08]	204/639	1.73 [1.36–2.19]	161/666	1.20 [0.94–1.53]	67/835	1.40 [1.00–1.98]
Moderate (50–79)	487/1017	1.39 [1.16–1.66]	377/1306	1.33 [1.09–1.61]	405/1310	1.31 [1.09–1.58]	163/1739	1.37 [1.05–1.79]
High (80–100)	119/263	1.19 [0.89–1.59]	111/351	1.55 [1.17–2.05]	104/340	1.28 [0.96–1.69]	59/473	1.87 [1.32–2.65]
Health factors score								
Low (0–49)	391/712	2.26 [1.86–2.75]	385/1043	2.15 [1.78–2.61]	352/1000	1.94 [1.61–2.35]	172/1560	1.74 [1.34–2.26]
Moderate (50–79)	394/889	1.42 [1.18–1.70]	270/1035	1.28 [1.05–1.57]	284/1091	1.25 [1.03–1.51]	106/1264	1.40 [1.05–1.87]
High (80–100)	62/207	0.77 [0.55–1.07]	37/218	0.80 [0.53–1.19]	34/225	0.64 [0.43–0.96]	11/223	0.86 [0.45–1.64]
Overall CVH score								
Low (0–49)	289/540	2.34 [1.88–2.91]	271/743	2.17 [1.76–2.68]	233/725	1.89 [1.52–2.34]	107/1098	1.65 [1.23–2.21]
Moderate (50–79)	528/1170	1.43 [1.21–1.70]	404/1447	1.40 [1.17–1.69]	416/1481	1.33 [1.12–1.59]	171/1832	1.47 [1.13–1.91]
High (80–100)	30/98	0.74 [0.46–1.18]	17/106	0.73 [0.42–1.28]	21/110	0.77 [0.46–1.28]	11/117	1.61 [0.83–3.13]

Abbreviations: BMI, body mass index; baPWV, brachial‐ankle pulse wave velocity; CVH, cardiovascular health; CI, confidence interval; OR, odds ratio; PP, pulse pressure.

^a^ 5225 participants (1937 with normal glucose regulation and 3288 with hyperglycemia) were included in the analysis.

^b^ Adjusted for baseline age, sex, current drinking status, and education attainment (less than high school or high school or greater). Individual cardiovascular health metrics were mutually adjusted. CVH behaviors metrics and health factors metrics were mutually adjusted. Normal glucose regulation was used as the reference group.

**FIGURE 2:**
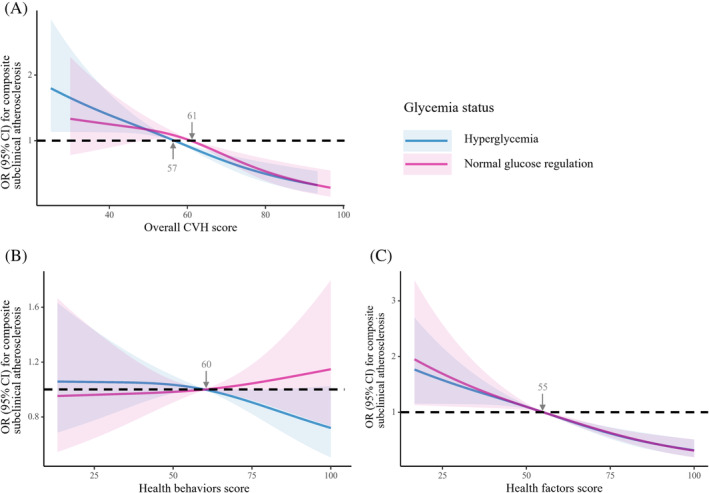
Associations between composite subclinical atherosclerosis and overall cardiovascular health (CVH) (A), health behaviors (B), or health factors (C) score according to glycemia status. Overall, 3220 participants (1412 with normal glucose regulation and 1808 with hyperglycemia) were included in the analysis. Solid lines are multivariable adjusted odds ratios (ORs), with shadows showing 95% confidence intervals (CIs). Reference lines for no association are indicated by the dashed black lines at an OR of 1.0. Arrows indicate the point of CVH score when the OR for composite subclinical atherosclerosis were 1.0. Analyses were adjusted for baseline age, sex, current drinking status, and education attainment (less than high school or high school or greater). CVH behaviors metrics and health factors metrics were mutually adjusted.

The association of higher scores of health factors or overall CVH with lower risk for composite subclinical atherosclerosis was consistent across normal glucose regulation and hyperglycemia strata (Figure 2). Among individuals with normal glucose regulation or hyperglycemia, the ORs of composite subclinical atherosclerosis for per 10‐point increment in baseline overall CVH were 0.80 (95% CI, 0.73–0.88) and 0.78 (95% CI, 0.72–0.84), and for per 10‐point increment in baseline scores of health factors were 0.80 (95% CI, 0.75–0.85) and 0.82 (95% CI, 0.77–0.86), respectively (Table 3). No statistically significant interaction was observed with glycemia status (*p*_int_ > 0.05). Similar results were observed in other investigated outcomes reflected by elevated baPWV, elevated PP, and albuminuria. Among participants with normal glucose regulation, the OR for composite subclinical atherosclerosis was 1.16 (95% CI, 0.75–1.80) for those who had high score of health behaviors compared with low score and was 1.02 (95% CI, 0.96–1.10) for per 10‐point increment in health behaviors. While, among individuals with hyperglycemia, the OR was 0.68 (95% CI, 0.48–0.97) for those who had high score of health behaviors compared with low score and was 0.95 (95% CI, 0.90–1.01) per 10‐point increment in health behaviors (Table 3). Adherence to healthy behaviors had a significant reduction in the burden of subclinical CVD among high‐risk individuals.

**TABLE 3:** Odds ratio (95% CI) of subclinical atherosclerosis according to cardiovascular health among participants with normal glucose regulation or hyperglycemia.^a^

Category	Composite subclinical atherosclerosis		Elevated baPWV		Elevated PP		Albuminuria	
	Case/*N*	OR (95% CI)^b^	Case/*N*	OR (95% CI)^b^	Case/*N*	OR (95% CI)^b^	Case/*N*	OR (95% CI)^b^
Normal glucose regulation								
Health behaviors score								
Low (0–49)	118/406	1.00 (ref)	84/446	1.00 (ref)	66/448	1.00 (ref)	27/512	1.00 (ref)
Moderate (50–79)	264/826	1.04 [0.75–1.44]	172/966	0.91 [0.63–1.30]	188/948	1.19 [0.82–1.71]	61/1094	0.76 [0.44–1.31]
High (80–100)	62/180	1.16 [0.75–1.80]	32/216	0.73 [0.44–1.21]	51/220	1.32 [0.83–2.11]	12/246	0.64 [0.30–1.36]
Per 10‐point increment	NA	1.02 [0.96–1.10]	NA	0.95 [0.88–1.03]	NA	1.05 [0.98–1.14]	NA	0.91 [0.80–1.03]
Health factors score								
Low (0–49)	165/364	1.00 (ref)	127/481	1.00 (ref)	131/450	1.00 (ref)	48/616	1.00 (ref)
Moderate (50–79)	218/717	0.51 [0.39–0.68]	131/808	0.54 [0.41–0.73]	146/818	0.53 [0.40–0.70]	41/892	0.62 [0.40–0.96]
High (80–100)	61/331	0.30 [0.21–0.43]	30/339	0.33 [0.21–0.52]	28/348	0.25 [0.16–0.39]	11/344	0.51 [0.26–1.01]
Per 10‐point increment	NA	0.80 [0.75–0.85]	NA	0.81 [0.75–0.88]	NA	0.77 [0.72–0.83]	NA	0.87 [0.78–0.97]
Overall CVH score								
Low (0–49)	99/273	1.00 (ref)	83/338	1.00 (ref)	71/328	1.00 (ref)	32/438	1.00 (ref)
Moderate (50–79)	320/1006	0.73 [0.53–1.00]	197/1153	0.62 [0.44–0.86]	218/1142	0.70 [0.50–0.98]	61/1271	0.58 [0.36–0.93]
High (80–100)	25/133	0.40 [0.23–0.70]	8/137	0.24 [0.11–0.54]	16/146	0.37 [0.20–0.70]	7/143	0.66 [0.27–1.60]
Per 10‐point increment	NA	0.80 [0.73–0.88]	NA	0.76 [0.68–0.85]	NA	0.80 [0.72–0.89]	NA	0.78 [0.66–0.93]
Hyperglycemia								
Health behaviors score								
Low (0–49)	241/528	1.00 (ref)	204/639	1.00 (ref)	161/666	1.00 (ref)	67/835	1.00 (ref)
Moderate (50–79)	487/1017	0.80 [0.61–1.04]	377/1306	0.76 [0.59–0.98]	405/1310	1.18 [0.91–1.53]	163/1739	0.98 [0.70–1.38]
High (80–100)	119/263	0.68 [0.48–0.97]	111/351	0.88 [0.63–1.23]	104/340	1.16 [0.83–1.62]	59/473	1.32 [0.87–1.99]
Per 10‐point increment	NA	0.95 [0.90–1.01]	NA	0.99 [0.94–1.04]	NA	1.02 [0.97–1.08]	NA	1.04 [0.97–1.11]
Health factors score								
Low (0–49)	391/712	1.00 (ref)	385/1043	1.00 (ref)	352/1000	1.00 (ref)	172/1560	1.00 (ref)
Moderate (50–79)	394/889	0.62 [0.51–0.77]	270/1035	0.60 [0.49–0.73]	284/1091	0.64 [0.53–0.78]	106/1264	0.80 [0.62–1.04]
High (80–100)	62/207	0.34 [0.24–0.48]	37/218	0.37 [0.25–0.55]	34/225	0.33 [0.22–0.50]	11/223	0.49 [0.26–0.92]
Per 10‐point increment	NA	0.82 [0.77–0.86]	NA	0.82 [0.78–0.87]	NA	0.83 [0.78–0.87]	NA	0.89 [0.82–0.95]
Overall CVH score								
Low (0–49)	289/540	1.00 (ref)	271/743	1.00 (ref)	233/725	1.00 (ref)	107/1098	1.00 (ref)
Moderate (50–79)	528/1170	0.58 [0.46–0.73]	404/1447	0.64 [0.52–0.79]	416/1481	0.71 [0.58–0.88]	171/1832	0.88 [0.67–1.15]
High (80–100)	30/98	0.29 [0.18–0.48]	17/106	0.33 [0.19–0.59]	21/110	0.41 [0.24–0.70]	11/117	0.97 [0.50–1.89]
Per 10‐point increment	NA	0.78 [0.72–0.84]	NA	0.81 [0.75–0.88]	NA	0.85 [0.78–0.91]	NA	0.92 [0.83–1.02]

Abbreviations: baPWV, brachial‐ankle pulse wave velocity; CVH, cardiovascular health; CI, confidence interval; NA, not applicable; OR, odds ratio; PP, pulse pressure.

^a^ 5225 participants (1937 with normal glucose regulation and 3288 with hyperglycemia) were included in the analysis.

^b^ Adjusted for baseline age, sex, current drinking status, and education attainment (less than high school or high school or greater). CVH behaviors metrics and health factor metrics were mutually adjusted.

**FIGURE 3:**
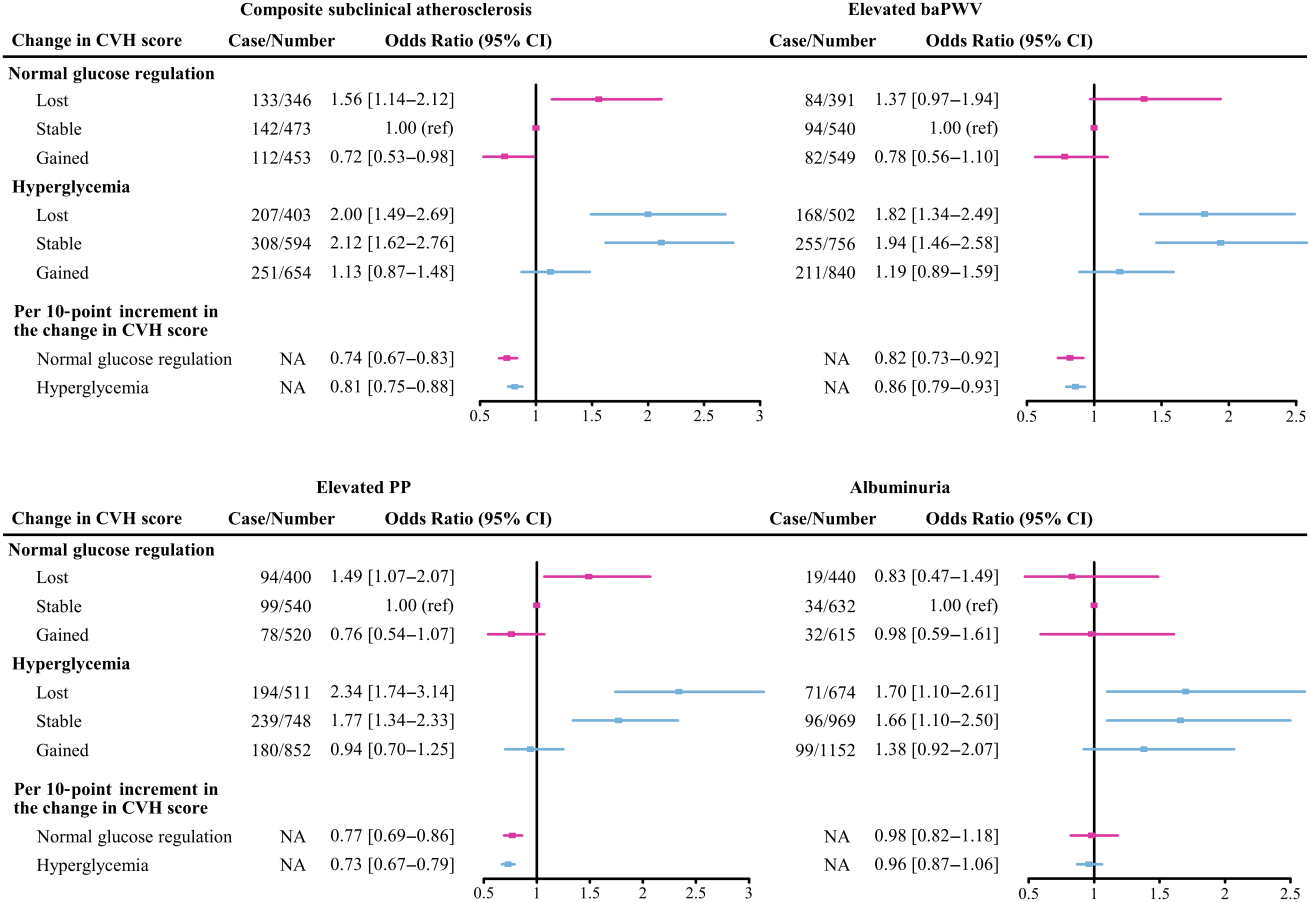
Change in the overall cardiovascular health (CVH) score between 2010 and 2014 and associations with subclinical atherosclerosis. Overall, 4768 participants with complete data on Life's Essential 8 metrics at follow‐up and without cardiovascular disease (CVD) events during 4.3 years follow‐up (1762 with normal glucose regulation and 3006 with hyperglycemia) were included in the analysis. The score, representing the change in overall CVH from 2010 to 2014, was categorized as gained in those with more than 5 points, stable in those with −5 to 5 points, lost in those with less than −5 points. Adjusted for baseline age, sex, current drinking status, and education attainment (less than high school or high school or greater). baPWV, brachial‐ankle pulse wave velocity; CI, confidence interval; NA, not applicable; PP, pulse pressure.

We next investigated whether the risk of subclinical atherosclerosis among individuals with different baseline glucose tolerance status was modified by the transition in overall CVH during follow‐up. As shown in Figure 3, risk for composite subclinical atherosclerosis was especially high in all groups of lost overall CVH score during follow‐up. Compared with normal glucose regulation and stable CVH group, the OR was 1.56 (95% CI, 1.14–2.12) for participants who had normal glucose regulation with lost CVH and 2.00 (95% CI, 1.49–2.69) for hyperglycemia with lost CVH. Meanwhile, participants with hyperglycemia and stable CVH also had an increased risk (OR, 2.12; 95% CI, 1.62–2.76). On the contrary, compared with normal glucose regulation and stable CVH group, the risk of composite subclinical atherosclerosis decreased 28% in those with normal glucose regulation and gained CVH (OR, 0.72; 95% CI, 0.53–0.98), but remained nonsignificantly higher in those with hyperglycemia and gained CVH (OR, 1.13; 95% CI, 0.87–1.48). Results did not change when we assessed the risk of elevated baPWV, elevated PP and albuminuria among individuals who had hyperglycemia and improved CVH. For each 10‐point increment in change of overall CVH score between 2010 and 2014, there was a 26% and 19% lower risk of composite subclinical atherosclerosis in persons with normal glucose regulation and hyperglycemia, respectively.

## 4 DISCUSSION

In this prospective cohort study, we indicated that the novel definition of CVH based on LE8 was inversely associated with subsequent risk of subclinical atherosclerosis and further demonstrated that CVH status and its change modified the association between hyperglycemia and subclinical atherosclerosis. Compared with low score of overall CVH, high score was associated with 60% and 71% lower risks of composite subclinical atherosclerosis among participants with normal glucose regulation and hyperglycemia, respectively. Participants with hyperglycemia who had high score of individual or overall CVH exhibited no significant excess risk of subclinical atherosclerosis compared with those with normal glucose regulation. Furthermore, we demonstrated that during a median follow‐up of 4.3 years, improvement in overall CVH showed a beneficial effect on reduced risk of composite subclinical atherosclerosis among individuals with hyperglycemia, compared to those who had normal glucose regulation and stable CVH.

To the best of our knowledge, this study is the first to comprehensively investigate the association of CVH score using new LE8 metrics with subclinical atherosclerosis among individuals with different baseline glucose tolerance status. We addressed a few major limitations in previous studies by: (1) using the LE8 algorithm to provide a broader and more granular description of CVH, (2) examining the early stage of CVD to help establish the earlier management of risk factors, and (3) evaluating the impact of change in overall CVH on the risk of subclinical atherosclerosis to adopt promotion of healthy behaviors or factors during midlife, not only primordially at early stages of life.

In the general population, it is well established that risk factor management and healthy lifestyle are associated with a lower risk of subclinical atherosclerosis.^22^, ^23^ For example, those who had the combination of three lifestyle behaviors (Mediterranean diet, nonsmoking, and moderate alcohol intake) were related to a 18% lower risk of subclinical atherosclerosis compared with their counterparts.^24^ It has been reported that tobacco cigarette smoke has severe cardiovascular side effects leading to endothelial dysfunction, increased oxidative stress, and increased cardiovascular morbidity and mortality,^25^ which would be stopped by nonsmoking. Yet regular physical activity can play an important role on a healthier metabolic milieu with attenuation of systemic chronic inflammation, as well as adaptations at the vascular (antiatherogenic effects) level.^26^ When metabolic risk factors and their upstream behavioral factors were combined together by taking advantage of comprehensive LS7 CVH metrics, an observational study indicated that the risks of increased carotid intima‐media thickness (CIMT) and increased baPWV have substantially reduced in participants who had five or more ideal metrics compared with those with only 0–1 ideal metric.^17^ Meanwhile, numerous epidemiological studies identified that sleep metric was an important factor influencing risk of subclinical atherosclerosis^27^ and even added independent predictive value for CVD events over and above the original seven CVH metrics.^28^, ^29^ Using the new LE8 CVH metrics, which contemplated the inclusion of sleep metric, the current study demonstrated that higher score of overall CVH was inversely associated with subclinical atherosclerosis.

Among people with hyperglycemia, regarding the associations of metabolic and behavioral factors with health outcomes, previous studies largely focused on the associations between adhering to CVH and clinical CVD events.^30^, ^31^, ^32^ Data linking subclinical atherosclerosis with CVH metrics were rare, and existing evidence for LE8 CVH in this regard is somewhat lacking. In the current study, participants with hyperglycemia exhibited no significant excess risk of subclinical atherosclerosis compared with those with normal glucose regulation, when the score of cardiovascular behaviorial factors, metabolic factors, or overall CVH was higher than 80. Nevertheless, given the fact that patients with hyperglycemia had a higher inherent risk of cardiovascular events,^33^ a higher CVH score did reduce the risk of subclinical atherosclerosis. Previous studies found that diabetes patients in multifactorial intensive intervention group with an ideal range of blood glucose, pressure and lipids showed significantly reduced formation of atherosclerotic plaques and even an overall regression of mean CIMT, which is consistent with our findings.^34^, ^35^ Nevertheless, our findings are not concordant with the results of the Diabetes Prevention Program, which found no difference in coronary calcium scores in the lifestyle intervention group receiving a low‐calorie, low‐fat diet, and moderate physical activity as compared with the placebo group among adults with prediabetes.^36^ The relatively short intervention or reduced efficacy as measured by a diminution of weight loss may at least partially explain the absence of an effect of lifestyle intervention. Overall, it is important to promote the adherence to better overall CVH in the prevention of subclinical CVD events among patients with prediabetes or diabetes.

Meanwhile, making healthy behavior or metabolic changes could alter risk of subclinical atherosclerosis. As shown in our study, each additional 10 points in the changes in overall CVH during a median 4.3 years of follow‐up was associated with a 26% and 19% reduction in risk of composite subclinical atherosclerosis among participants with normal glucose regulation and hyperglycemia, respectively. Moreover, improvements in CVH could partly counteract the higher risk of subclinical atherosclerosis among participants with hyperglycemia. Our findings are consistent with previous observations in a pooled cohort study of 9388 individuals, where groups experienced more rapid declines in scores from four of seven CVH metrics—BMI, BP, TC, and blood glucose level‐during childhood and adolescence—were more likely to have a higher burden of elevated CIMT by middle age.^37^ Preserving and promoting CVH from early life onward appears to be an effective way to reduce CVD risk later in life. Besides, in the Coronary Artery Risk Development in Young Adults (CARDIA) prospective cohort study, change in five healthy lifestyle factors (not overweight/obese, low alcohol intake, healthy diet, physically active, and nonsmoker) during young adulthood is linked to the decreased burden of coronary artery calcification and CIMT in middle age.^38^ Collectively, promotion of overall CVH is essential among elderly and middle‐aged adults to control and potentially even reverse the progression of subclinical atherosclerosis.

With regard to each component of CVH metrics, a high BP score was predominantly associated with lower risks of subclinical atherosclerosis among individuals with hyperglycemia. BP is an important determinant of the CVD risks. Approximately 23% of the population‐attributable fractions for CVD were attributed to the hypertension among 12 metabolic, behavioral, and psychosocial risk factors.^39^ The risk of major cardiovascular events has decreased 6% in participants with diabetes when there was a 5 mmHg reduction in SBP based on a meta‐analysis of 51 randomized clinical trials.^40^ Besides, the results of the Action in Diabetes and Vascular disease: preterAx and diamicroN‐MR Controlled Evaluation (ADVANCE) trial indicate that the routine administration of a fixed combination of perindopril and indapamide to a broad range of patients with diabetes reduce the risks of microvascular complications, although with wide confidence limits.^41^ However, the proportion of BP being undertreated was quite high among individuals with diabetes.^42^ These gaps provided significant opportunities to strengthen the control of BP at high‐risk population and more cardiovascular complication would be avoided.^43^ Besides, targeting both lifestyle behaviors and metabolic profiles among individuals with hyperglycemia was important for the prevention of subclinical atherosclerosis. For example, in the Look AHEAD (Action for Health in Diabetes) trial, only focusing on an intensive lifestyle intervention that promoted weight loss through decreased caloric intake and increased physical activity did not mitigate the risk of cardiovascular morbidity among patients with diabetes.^44^ In contrast, taking advantage of comprehensive LS7 CVH, five or more ideal metrics were associated with 58% and 61% lower CVD risks compared with one or none ideal metric among participants with prediabetes and diabetes, respectively.^18^ Overall, our findings and existing evidence suggest that an overall healthy behaviors and factors can significantly aid in the prevention of CVD among patients with hyperglycemia.

The strengths of this study included the prospective study design, the updated definitions of CVH, the evaluation of changes in CVH over time, and comprehensive analyses including individuals with different baseline glucose tolerance status. However, our study has some limitations. First, dietary data were not available to calculate the complete CVH score. However, because the benefit of ideal CVH is graded,^45^ the relationship between LE8 CVH and atherosclerosis would not be skewed or reversed by missing one of CVH metrics. Second, elevated baPWV, PP, and albuminuria, considered as the investigated outcomes, are surrogate indicators of cardiovascular events. While, the strong associations of the above markers with subsequent CVD risks and a combination of these key indicators in the present study might reduce such gap.^13^, ^14^, ^15^ Third, the median follow‐up duration of 4.3 years was relatively short and might be insufficient to uncover the CVH transition and the incidence of events. More well powered epidemiological studies with a longer period of follow‐up and completed CVH metrics are needed to help clarify this issue.

In conclusion, patients with hyperglycemia who achieved a high score of overall CVH exhibited no significant excess risks of subclinical atherosclerosis compared with individuals with normal glucose regulation. In addition, improvements in overall CVH during a median follow‐up of 4.3 years were also associated with a lower risk of subsequent subclinical atherosclerosis. Our findings support the importance of promoting the adherence to, and the improvement of, both healthy behaviors and factors as a key strategy to tackle the onset of atherosclerosis and reduce CVD burden among patients with diabetes or prediabetes.

## AUTHOR CONTRIBUTIONS

XJ, CH, HL, GN, YB, and JLL designed the study. XJ, YD, and CH performed the data analysis. XJ drafted the manuscript. YD, LL, XW, HQ, SW, RZ, JZ, MX, YX, TW, ZZ, YC, ML, WW, and WH revised the manuscript critically for important intellectual content. All authors were involved in writing and revising the paper and had final approval of the submitted and published versions.

## CONFLICT OF INTEREST STATEMENT

GN, WW, and YB are Editorial Board members of *Journal of Diabetes* and co‐authors of this article. To minimize bias, they were excluded from all editorial decision‐making related to the acceptance of this article for publication.

## Supporting information


**Data S1.** Supporting Information.

## References

[1] Lloyd‐Jones DM , Hong Y , Labarthe D , et al. Defining and setting national goals for cardiovascular health promotion and disease reduction: the American Heart Association's strategic impact goal through 2020 and beyond. Circulation. 2010;121(4):586‐613. 20089546 10.1161/CIRCULATIONAHA.109.192703

[2] Dong C , Rundek T , Wright CB , Anwar Z , Elkind MS , Sacco RL . Ideal cardiovascular health predicts lower risks of myocardial infarction, stroke, and vascular death across whites, blacks, and hispanics: the Northern Manhattan study. Circulation. 2012;125(24):2975‐2984. 22619283 10.1161/CIRCULATIONAHA.111.081083 PMC3396556

[3] Lloyd‐Jones DM , Allen NB , Anderson CAM , et al. Life's essential 8: updating and enhancing the American Heart Association's construct of cardiovascular health: a presidential advisory from the American Heart Association. Circulation. 2022;146(5):e18‐e43. 35766027 10.1161/CIR.0000000000001078 PMC10503546

[4] Lloyd‐Jones DM , Ning H , Labarthe D , et al. Status of cardiovascular health in US adults and children using the American Heart Association's new "Life's essential 8" metrics: prevalence estimates from the National Health and nutrition examination survey (NHANES), 2013 through 2018. Circulation. 2022;146(11):822‐835. 35766033 10.1161/CIRCULATIONAHA.122.060911

[5] International Diabetes Federation . IDF Diabetes Atlas. 10th edition. Accessed October 31, 2022. http://www.diabetesatlas.org/

[6] Rao Kondapally Seshasai S , Kaptoge S , Thompson A , et al. Diabetes mellitus, fasting glucose, and risk of cause‐specific death. N Engl J Med. 2011;364(9):829‐841. 21366474 10.1056/NEJMoa1008862 PMC4109980

[7] Barr EL , Zimmet PZ , Welborn TA , et al. Risk of cardiovascular and all‐cause mortality in individuals with diabetes mellitus, impaired fasting glucose, and impaired glucose tolerance: the Australian Diabetes, Obesity, and Lifestyle Study (AusDiab). Circulation. 2007;116(2):151‐157. 17576864 10.1161/CIRCULATIONAHA.106.685628

[8] Cai X , Zhang Y , Li M , et al. Association between prediabetes and risk of all cause mortality and cardiovascular disease: updated meta‐analysis. BMJ. 2020;370:m2297. 32669282 10.1136/bmj.m2297 PMC7362233

[9] Huang Y , Cai X , Chen P , et al. Associations of prediabetes with all‐cause and cardiovascular mortality: a meta‐analysis. Ann Med. 2014;46(8):684‐692. 25230915 10.3109/07853890.2014.955051

[10] Sarwar N , Gao P , Seshasai SR , et al. Diabetes mellitus, fasting blood glucose concentration, and risk of vascular disease: a collaborative meta‐analysis of 102 prospective studies. Lancet. 2010;375(9733):2215‐2222. 20609967 10.1016/S0140-6736(10)60484-9 PMC2904878

[11] Rubin J , Nambi V , Chambless LE , et al. Hyperglycemia and arterial stiffness: the atherosclerosis risk in the communities study. Atherosclerosis. 2012;225(1):246‐251. 23031361 10.1016/j.atherosclerosis.2012.09.003 PMC3936879

[12] Xanthakis V , Sung JH , Samdarshi TE , et al. Relations between subclinical disease markers and type 2 diabetes, metabolic syndrome, and incident cardiovascular disease: the Jackson heart study. Diabetes Care. 2015;38(6):1082‐1088. 25765357 10.2337/dc14-2460 PMC4439537

[13] Ohkuma T , Ninomiya T , Tomiyama H , et al. Brachial‐ankle pulse wave velocity and the risk prediction of cardiovascular disease: an individual participant data meta‐analysis. Hypertension. 2017;69(6):1045‐1052. 28438905 10.1161/HYPERTENSIONAHA.117.09097

[14] Franklin SS , Khan SA , Wong ND , Larson MG , Levy D . Is pulse pressure useful in predicting risk for coronary heart disease? The Framingham Heart Study. Circulation. 1999;100(4):354‐360. 10421594 10.1161/01.cir.100.4.354

[15] Jørgensen L , Jenssen T , Johnsen SH , et al. Albuminuria as risk factor for initiation and progression of carotid atherosclerosis in non‐diabetic persons: the Tromsø Study. Eur Heart J. 2007;28(3):363‐369. 17132646 10.1093/eurheartj/ehl394

[16] Xanthakis V , Enserro DM , Murabito JM , et al. Ideal cardiovascular health: associations with biomarkers and subclinical disease and impact on incidence of cardiovascular disease in the Framingham Offspring Study. Circulation. 2014;130(19):1676‐1683. 25274000 10.1161/CIRCULATIONAHA.114.009273

[17] Wang L , Niu JY , Zhao ZY , et al. Ideal cardiovascular health is inversely associated with subclinical atherosclerosis: a prospective analysis. Biomed Environ Sci. 2019;32(4):260‐271. 31217062 10.3967/bes2019.036

[18] Wang T , Lu J , Su Q , et al. Ideal cardiovascular health metrics and major cardiovascular events in patients with prediabetes and diabetes. JAMA Cardiol. 2019;4(9):874‐883. 31365039 10.1001/jamacardio.2019.2499 PMC6669896

[19] Lin L , Zhang J , Jiang L , et al. Transition of metabolic phenotypes and risk of subclinical atherosclerosis according to BMI: a prospective study. Diabetologia. 2020;63(7):1312‐1323. 32130460 10.1007/s00125-020-05116-5

[20] Craig CL , Marshall AL , Sjöström M , et al. International Physical Activity Questionnaire: 12‐country reliability and validity. Med Sci Sports Exerc. 2003;35(8):1381‐1395. 12900694 10.1249/01.MSS.0000078924.61453.FB

[21] Diagnosis and classification of diabetes mellitus. Diabetes Care. 2010;33(Suppl 1):S62‐S69. 20042775 10.2337/dc10-S062 PMC2797383

[22] Nissen SE , Nicholls SJ , Sipahi I , et al. Effect of very high‐intensity statin therapy on regression of coronary atherosclerosis: the ASTEROID trial. JAMA. 2006;295(13):1556‐1565. 16533939 10.1001/jama.295.13.jpc60002

[23] Wang D , Jackson EA , Karvonen‐Gutierrez CA , et al. Healthy lifestyle during the midlife is prospectively associated with less subclinical carotid atherosclerosis: the study of women's health across the nation. J Am Heart Assoc. 2018;7(23):e010405. 30482079 10.1161/JAHA.118.010405 PMC6405552

[24] Uzhova I , Mateo‐Gallego R , Moreno‐Franco B , et al. The additive effect of adherence to multiple healthy lifestyles on subclinical atherosclerosis: insights from the AWHS. J Clin Lipidol. 2018;12(3):615‐625. 29680699 10.1016/j.jacl.2018.03.081

[25] Münzel T , Hahad O , Kuntic M , Keaney JF Jr , Deanfield JE , Daiber A . Effects of tobacco cigarettes, e‐cigarettes, and waterpipe smoking on endothelial function and clinical outcomes. Eur Heart J. 2020;41(41):4057‐4070. 32585699 10.1093/eurheartj/ehaa460 PMC7454514

[26] Valenzuela PL , Ruilope LM , Santos‐Lozano A , et al. Exercise benefits in cardiovascular diseases: from mechanisms to clinical implementation. Eur Heart J. 2023;44(21):1874‐1889. 37005351 10.1093/eurheartj/ehad170

[27] Domínguez F , Fuster V , Fernández‐Alvira JM , et al. Association of sleep duration and quality with subclinical atherosclerosis. J Am Coll Cardiol. 2019;73(2):134‐144. 30654884 10.1016/j.jacc.2018.10.060

[28] Makarem N , Castro‐Diehl C , St‐Onge MP , et al. Abstract 36: The role of sleep as a cardiovascular health metric: does it improve cardiovascular disease risk prediction? Results from the Multi‐Ethnic Study of Atherosclerosis. Circulation. 2020;141:A36. doi:10.1161/circ.141.suppl_1.36

[29] Makarem N , Castro‐Diehl C , St‐Onge MP , et al. Redefining cardiovascular health to include sleep: prospective associations with cardiovascular disease in the MESA sleep study. J Am Heart Assoc. 2022;11(21):e025252. 36259552 10.1161/JAHA.122.025252 PMC9673642

[30] Gaede P , Lund‐Andersen H , Parving HH , Pedersen O . Effect of a multifactorial intervention on mortality in type 2 diabetes. N Engl J Med. 2008;358(6):580‐591. 18256393 10.1056/NEJMoa0706245

[31] Han H , Cao Y , Feng C , et al. Association of a healthy lifestyle with all‐cause and cause‐specific mortality among individuals with type 2 diabetes: a prospective study in UK Biobank. Diabetes Care. 2022;45(2):319‐329. 34857534 10.2337/dc21-1512

[32] Li G , Zhang P , Wang J , et al. Cardiovascular mortality, all‐cause mortality, and diabetes incidence after lifestyle intervention for people with impaired glucose tolerance in the Da Qing Diabetes Prevention Study: a 23‐year follow‐up study. Lancet Diabetes Endocrinol. 2014;2(6):474‐480. 24731674 10.1016/S2213-8587(14)70057-9

[33] Ferket BS , Hunink MGM , Masharani U , et al. Lifetime cardiovascular disease risk by coronary artery calcium score in individuals with and without diabetes: an analysis from the multi‐ethnic study of atherosclerosis. Diabetes Care. 2022;45(4):975‐982. 35168253 10.2337/dc21-1607 PMC9114718

[34] Shi C , Men L , Yu C , et al. Atherosclerosis associated with dynamic inflammation changes after multifactorial intervention in short‐duration type 2 diabetes: a randomized, controlled, 10‐year follow‐up trial. J Diabetes Complications. 2017;31(8):1286‐1292. 28610945 10.1016/j.jdiacomp.2017.05.008

[35] Tripolt NJ , Narath SH , Eder M , Pieber TR , Wascher TC , Sourij H . Multiple risk factor intervention reduces carotid atherosclerosis in patients with type 2 diabetes. Cardiovasc Diabetol. 2014;13:95. 24884694 10.1186/1475-2840-13-95 PMC4041351

[36] Goldberg RB , Aroda VR , Bluemke DA , et al. Effect of long‐term metformin and lifestyle in the diabetes prevention program and its outcome study on coronary artery calcium. Circulation. 2017;136(1):52‐64. 28476766 10.1161/CIRCULATIONAHA.116.025483 PMC5526695

[37] Allen NB , Krefman AE , Labarthe D , et al. Cardiovascular health trajectories from childhood through middle age and their association with subclinical atherosclerosis. JAMA Cardiol. 2020;5(5):557‐566. 32159727 10.1001/jamacardio.2020.0140 PMC7066520

[38] Spring B , Moller AC , Colangelo LA , et al. Healthy lifestyle change and subclinical atherosclerosis in young adults: coronary artery risk development in young adults (CARDIA) study. Circulation. 2014;130(1):10‐17. 24982115 10.1161/CIRCULATIONAHA.113.005445 PMC4615574

[39] Walli‐Attaei M , Rosengren A , Rangarajan S , et al. Metabolic, behavioural, and psychosocial risk factors and cardiovascular disease in women compared with men in 21 high‐income, middle‐income, and low‐income countries: an analysis of the PURE study. Lancet. 2022;400(10355):811‐821. 36088949 10.1016/S0140-6736(22)01441-6

[40] Nazarzadeh M , Bidel Z , Canoy D , et al. Blood pressure‐lowering treatment for prevention of major cardiovascular diseases in people with and without type 2 diabetes: an individual participant‐level data meta‐analysis. Lancet Diabetes Endocrinol. 2022;10(9):645‐654. 35878651 10.1016/S2213-8587(22)00172-3 PMC9622419

[41] Patel A , MacMahon S , Chalmers J , et al. Effects of a fixed combination of perindopril and indapamide on macrovascular and microvascular outcomes in patients with type 2 diabetes mellitus (the ADVANCE trial): a randomised controlled trial. Lancet. 2007;370(9590):829‐840. 17765963 10.1016/S0140-6736(07)61303-8

[42] Pagidipati NJ , Navar AM , Pieper KS , et al. Secondary prevention of cardiovascular disease in patients with type 2 diabetes mellitus: international insights from the TECOS trial (Trial Evaluating Cardiovascular Outcomes With Sitagliptin). Circulation. 2017;136(13):1193‐1203. 28626088 10.1161/CIRCULATIONAHA.117.027252 PMC5614823

[43] Yu Y , Sun Y , Yu Y , et al. Life's essential 8 and risk of non‐communicable chronic diseases: outcome‐wide analyses. Chin Med J (Engl). 2023;137:1553‐1562. 37821910 10.1097/CM9.0000000000002830 PMC11230768

[44] Wing RR , Bolin P , Brancati FL , et al. Cardiovascular effects of intensive lifestyle intervention in type 2 diabetes. N Engl J Med. 2013;369(2):145‐154. 23796131 10.1056/NEJMoa1212914 PMC3791615

[45] Hadaegh F , Hosseinpour‐Niazi S , Deravi N , et al. Ideal cardiovascular health status and risk of cardiovascular disease and all‐cause mortality: over a decade of follow‐up in the Tehran lipid and glucose study. Front Cardiovasc Med. 2022;9:898681. 35990976 10.3389/fcvm.2022.898681 PMC9386047

