# Supplementary material for: The association of ideal cardiovascular health and its change with subclinical atherosclerosis according to glucose status: A prospective cohort study
Source: J Diabetes. 2024 Oct 10;16(10):e70007. doi: 10.1111/1753-0407.70007 (PMC11464993; doi:10.1111/1753-0407.70007)
Supplement: Supplementary file 1 — Data S1. Supporting Information. [file JDB-16-e70007-s001.docx]

**Supplementary material**

**Supplementary Table 1.** Definitions of score of Life's Essential 8 CVH metric in Chinese adults aged ≥20 years

| **Domain** | **CVH metric** | **Quantification of CVH metric** | | |
| --- | --- | --- | --- | --- |
|  |  | **Description** | **Quantification** | **Points** |
| Health behaviors | PA | Minutes of moderate- (or greater) intensity activity per week. | >150 | 100 |
|  |  |  | 120–149 | 90 |
|  |  |  | 90–119 | 80 |
|  |  |  | 60–89 | 60 |
|  |  |  | 30–59 | 40 |
|  |  |  | 1–29 | 20 |
|  |  |  | 0 | 0 |
|  | Nicotine  exposure | Combustible tobacco use or secondhand smoke exposure. | Never smoker | 100 |
|  |  |  | Former smoker, quit ≥5 y | 75 |
|  |  |  | Former smoker, quit 1–<5 y | 50 |
|  |  |  | Former smoker, quit <1 y | 25 |
|  |  |  | Current smoker | 0 |
|  |  |  | Subtract 20 points (unless score is 0) for living with active indoor smoker in home. | |
|  | Sleep health | Self-reported average hours of sleep per night. | 7–<9 | 100 |
|  |  |  | 9–<10 | 90 |
|  |  |  | 6–<7 | 70 |
|  |  |  | 5–<6 or ≥10 | 40 |
|  |  |  | 4–<5 | 20 |
|  |  |  | <4 | 0 |
| Health factors | BMI | Body weight (kilograms) divided by height squared (meters squared) (kg/m^2^). | 18.5–22.9 | 100 |
|  |  |  | 23.0–24.9 | 75 |
|  |  |  | 25.0–29.9 | 50 |
|  |  |  | 30.0–34.9 | 25 |
|  |  |  | ≥35.0 | 0 |
|  | Blood lipids | Non-HDL-C (mg/dL). | <130 | 100 |
|  |  |  | 130–159 | 60 |
|  |  |  | 160–189 | 40 |
|  |  |  | 190–219 | 20 |
|  |  |  | ≥220 | 0 |
|  |  |  | If drug-treated level, subtract 20 points. | |
|  | BP | Systolic and diastolic BPs (mmHg). | <120/<80 (optimal) | 100 |
|  |  |  | 120–129/<80 (elevated) | 75 |
|  |  |  | 130–139 or 80–89 (stage 1 hypertension) | 50 |
|  |  |  | 140–159 or 90–99 | 25 |
|  |  |  | ≥160 or ≥100 | 0 |
|  |  |  | Subtract 20 points if treated level. | |

**Abbreviations:** BMI, body mass index; BP, blood pressure; CVH, cardiovascular health; non-HDL-C, non-high-density lipoprotein cholesterol; PA, physical activity.
